# A 95 year-old suffering circulatory arrest after accidental hypothermia: a case report

**DOI:** 10.1186/s12877-017-0646-6

**Published:** 2017-10-26

**Authors:** Anders Wetting Carlsen, Anders M. Winnerkvist, Guri Greiff

**Affiliations:** 1 Department of Cardiothoracic Anesthesiology and Intensive Care, St.Olavs Hospital, Trondheim University Hospital, Trondheim, Norway; 2 Department of Cardiothoracic Surgery, St.Olavs Hospital, Trondheim University Hospital, Trondheim, Norway; 3 Department of Circulation and Medical Imaging, Faculty of Medicine, NTNU, Norwegian University of Science and Technology, Trondheim, Norway

**Keywords:** Accidental hypothermia, Resuscitation, Extracorporeal life support, Cardiopulmonary bypass, Extracorporeal circulation

## Abstract

**Background:**

The elderly are vulnerable to cold and prone to accidental hypothermia, both because of environmental and endogenous factors. The incidence of severe accidental hypothermia among the elderly is poorly described, but many cases probably go unrecorded. Going through literature one finds few publications on severe hypothermia among the elderly, and, to our knowledge, nothing about extracorporeal re-warming of geriatric hypothermia victims.

**Case presentation:**

We present a case were a 95 year-old man with severe accidental hypothermia and circulatory arrest was brought to our hospital under on-going CPR, and was successfully resuscitated with extracorporeal circulation. He was discharged to his home without physical sequelae a few weeks later.

**Conclusion:**

The decision whether or not to continue resuscitation of a nonagenarian can be difficult in many respects. Knowing that resuscitation with extracorporeal circulation is resource intensive may complicate the discussion. In light of our experience with this case we discuss medical and ethical aspects of modern treatment of severe accidental hypothermia.

## Background

Re-warming with the use of Extracorporeal Circulation (ECC) is considered gold standard when treating victims of severe accidental hypothermia with concomitant unstable circulation or circulatory arrest [1–3]. An important prerequisite for successful treatment with a good neurological outcome is that the brain cools while the patient still breaths spontaneously before onset of cardiac arrest. In this way, brain oxygen consumption is decreased while oxygenation is maintained, making it possible for the brain to survive for a while even if the patient is clinically dead [4, 5].

Environmental and/or endogenous factors leave the elderly vulnerable to cold and prone to accidental hypothermia [6]. In spite of this, literature is scarce on the subject; little or nothing has been published regarding accidental hypothermia of the elderly and treatment using modern strategies and modalities.

We present a case where an elderly man with accidental hypothermia and circulatory arrest was brought to our hospital and resuscitated successfully on ECC.

## Case presentation

An elderly man with unknown identity was found soaking wet and cold in a basement, presumably after having tried to deal with a water leakage. When Para-Medics arrived on site he was awake, but restless and gave no adequate response. Initial ECG in the ambulance at 09:59 showed sinus rhythm with a heart rate of 50 pr. minute. At 10:01 the patient went into cardiac arrest, ECG showing ventricular fibrillation. CPR was started immediately and was continued during the short transport to hospital.

The patient was admitted to hospital at 10:03 under on-going resuscitation and was intubated shortly after arrival. During chest compressions he opened his eyes and raised his arms, implying very efficient CPR. On admittance his nasopharyngeal temperature was 22.9 °C. Initial blood gas analysis in the ER showed pH 6.9, s-Potassium 5.5 mmol/l and s-Lactate 12.5 mmol/l. In general, the criteria for ECC re-warming include core temperature < 32 °C and s-Potassium <8 mmol/l. Although the patient’s identity, age and previous medical history were unknown at the time, a swift decision was made to try to re-warm him with a Cardiopulmonary Bypass (CPB) machine. He was brought to the OR and re-warming with extracorporeal circulation was started at 10:42 after surgical cut-down and cannulation in the right femoral artery and vein. The patient was warmed slowly and was defibrillated into sinus rhythm after reaching a nasopharyngeal temperature of 31 °C. He was warmed further to 36 °C before being weaned successfully from CPB and was transferred sedated and intubated to the ICU.

At this point we finally received the patient’s identity. We were all a bit surprised to learn that he was a 95 year-old man with no previous medical history.

In the ICU the patient was hemodynamically and respiratory stable. He had flail chest and a unilateral pneumothorax due to multiple rib fractures after the chest compressions, but was extubated successfully the same afternoon. A chest tube was inserted and he received intermittent CPAP/BiPAP the first 36 h after extubation. Post-resuscitation chest pain was handled efficiently with thoracic epidural analgesia, and the patient was discharged from the ICU after two days. The postoperative course was uncomplicated, and after 13 days in hospital the patient was transferred to a nursing home for further rehabilitation. He regained his physical health quickly and was discharged to his home in his baseline status after three weeks of rehabilitation. He died from an unrelated cause almost three years later. Right up until his death he lived alone in his apartment with help from a home nurse service.

## Discussion and conclusion

On arrival in the ER we were skeptical about extracorporeal re-warming because of the patient’s age. After a brief bedside assessment his age was (wrongly) estimated to about 80 years. Considering other factors like short resuscitation time, relatively low s-Potassium and presumably very efficient chest compressions, we nevertheless chose to give him the benefit of doubt. In retrospect we are uncertain what our decision would have been if we had known on admission that he was 95 years old, but withdrawing resuscitation attempts would have been a likely option.

In a large registry of out-of-hospital cardiac arrests, survival to discharge for nonagenarians was only 2% [4, 7]. The pathophysiology of accidental hypothermia is of course different from other forms of cardiac arrest, and given the outcome of our patient one could argue that age alone should not be the only criterion for dismissing a patient from resuscitation on ECC. We had very little information about our patient, but if available, factors such as presence of major comorbidities, degree of autonomy, frailty, quality of life and mental status, should also be taken into consideration.

## Abbreviations

BiPAP: Bilevel Positive Airway Pressure
CPAP: Continuous Positive Airway Pressure
CPB: Cardiopulmonary Bypass
CPR: Cardiopulmonary Resuscitation
ECC: Extracorporeal Circulation
ER: Emergency Room
ICU: Intensive Care Unit
OR: Operating Room
VF: Ventricular Fibrillation
